# Efficient Removal of Carbamazepine from Synthetic Wastewater Using Potato Peel-Derived Hydrochars: A Comparative Study of Hydrothermal and Pyrolytic Conversion

**DOI:** 10.3390/molecules31132222

**Published:** 2026-06-24

**Authors:** Justin Khong, Bo Xiao, Chirangano Mangwandi

**Affiliations:** School of Chemistry & Chemical Engineering, Queen’s University Belfast, David Kier Building, Stranmillis Road, Belfast BT95AG, UK; jkhong01@qub.ac.uk (J.K.); b.xiao@qub.ac.uk (B.X.)

**Keywords:** adsorption, hydrochar, pharmaceutical contaminants, waste valorisation, wastewater treatment, circular economy

## Abstract

The increasing occurrence of pharmaceutical contaminants in aquatic environments has intensified the demand for sustainable and cost-effective water treatment technologies. This study investigated the conversion of potato peel waste into carbonaceous adsorbents through hydrothermal carbonization (HTC) and conventional pyrolysis (PRYR) for the removal of carbamazepine (CBZ) from synthetic wastewater. Hydrochars and biochars were synthesized under varying processing conditions and characterized using scanning electron microscopy (SEM), Fourier-transform infrared spectroscopy (FTIR), elemental analysis, and Brunauer–Emmett–Teller (BET) surface area analysis. Adsorption experiments were conducted using a 50 mg/L CBZ solution at pH 6, room temperature, and an adsorbent dosage of 1 g/L. The adsorption performance was evaluated after short contact times to assess rapid-removal capability. HTC-derived hydrochars exhibited significantly superior performance compared with pyrolysis-derived biochars, achieving up to 97% CBZ removal and adsorption capacities approaching 50 mg g^−1^ within 1 min of contact. In contrast, pyrolysis-derived biochars achieved removal efficiencies between approximately 7 and 55% under similar conditions. Correlation analysis between adsorption behaviour and physicochemical properties revealed that adsorption performance was more strongly influenced by surface chemistry, aromaticity, and mesoporosity than by BET surface area alone. FTIR analysis suggested that hydrogen bonding, π–π electron donor–acceptor interactions, and pore filling contributed to CBZ adsorption. HTC hydrochars retained abundant oxygen-containing functional groups that promoted rapid and stable adsorption, whereas pyrolysis-derived biochars exhibited weaker adsorption interactions despite possessing higher surface areas. The findings demonstrate that hydrothermal carbonization provides an effective low-temperature route for valorising potato peel waste into efficient adsorbents for rapid pharmaceutical removal from water and highlight the critical role of adsorbent surface chemistry in determining adsorption performance.

## 1. Introduction

The global transition toward a circular, resource-efficient, and climate-resilient economy has intensified interest in the valorization of biomass residues and organic wastes into energy carriers and value-added materials [1,2]. Contemporary waste management strategies increasingly emphasize the recovery of materials, chemicals, and energy from waste streams in alignment with the United Nations Sustainable Development Goals (SDGs), particularly SDG 6 (Clean Water and Sanitation), SDG 12 (Responsible Consumption and Production), and SDG 13 (Climate Action) [3,4]. Globally, approximately 1.3 billion tonnes of food waste are generated annually, representing nearly one-third of all food produced for human consumption [5]. Agricultural and food-processing residues constitute a substantial fraction of this waste stream and are increasingly recognized as promising renewable feedstocks for sustainable material production [6].

Among these residues, potato peel waste is generated in significant quantities from domestic consumption and agro-industrial processing. The global potato industry processes millions of tonnes of potatoes annually, producing peel residues that may account for approximately 15–40% of the total processed biomass, depending on the processing method [7,8]. Disposal of these residues presents environmental and logistical challenges due to their high moisture content, rapid biodegradability, and associated greenhouse gas emissions during decomposition [1,8]. Nevertheless, potato peel waste contains abundant lignocellulosic components, starch, proteins, and nitrogen-containing biomolecules, making it an attractive precursor for thermochemical conversion into functional carbonaceous materials [7,9]. Valorization of potato peel waste therefore offers a sustainable route for transforming low-value residue into environmentally beneficial adsorbent materials.

Thermochemical conversion technologies such as pyrolysis and HTC have been extensively investigated for biomass valorization. Conventional pyrolysis generally operates at temperatures between 300 and 900 °C under inert atmospheric conditions and produces biochars with enhanced aromaticity, carbon content, and pore development [10,11,12]. Chemical activation using agents such as KOH is frequently employed to further increase surface area and adsorption performance [12]. However, pyrolysis often requires energy-intensive drying of wet feedstocks and may reduce oxygen-containing functional groups that are beneficial for adsorption applications.

In contrast, hydrothermal carbonization is a low-temperature, water-mediated thermochemical process that commonly operates between 180 and 280 °C under autogenous pressure in sealed reactors [13,14,15]. HTC is particularly advantageous for wet biomass residues because it eliminates the need for prior drying while preserving oxygen- and nitrogen-containing surface functionalities [13,16]. During HTC, hydrolysis, dehydration, decarboxylation, and polymerization reactions contribute to the formation of hydrochars with tunable physicochemical properties [14,17]. Compared with pyrolysis, HTC generally requires lower operating temperatures and may therefore offer lower energy demand and improved sustainability for food-waste valorization [15,16].

One promising application of biomass-derived carbon materials is the adsorption-based removal of pharmaceutical contaminants from water systems. Pharmaceuticals are increasingly recognized as contaminants of emerging concern because of their persistence, bioaccumulation potential, and ecological impacts [3,18]. Carbamazepine (CBZ), a widely prescribed anticonvulsant and psychiatric medication, is among the most frequently detected pharmaceuticals in wastewater effluents, surface waters, and even drinking water supplies due to its resistance to biodegradation and conventional wastewater treatment processes [18,19]. Studies have reported CBZ concentrations ranging from ng L^−1^ to μg L^−1^ levels in aquatic environments worldwide, highlighting the need for efficient removal technologies [19,20].

Conventional approaches for CBZ removal include activated sludge treatment, membrane filtration, advanced oxidation processes, ozonation, and adsorption using activated carbon [19,20,21,22]. However, biological treatment methods often exhibit limited CBZ degradation efficiency due to the compound’s stable aromatic structure [19]. Membrane and oxidation technologies can achieve high removal efficiencies but are associated with high operational costs, membrane fouling, energy demand, and possible formation of toxic transformation products [21,22]. Adsorption using carbonaceous materials remains one of the most promising alternatives because of its simplicity, scalability, and effectiveness for micropollutant removal [23].

Previous studies have demonstrated successful CBZ adsorption using activated carbons, modified biochars, sludge-derived carbons, algae-based materials, and engineered composites [22,23,24,25,26,27,28]. In many cases, high adsorption capacities are associated with extensive chemical activation, high-temperature treatment, or complex modification strategies that may compromise economic feasibility and sustainability [27,28]. Pyrolysis-derived biochars typically exhibit a high surface area and aromaticity, promoting pore-filling and π–π interactions with aromatic pharmaceutical compounds [23,29]. Conversely, HTC-derived hydrochars generally possess lower surface areas but higher concentrations of oxygenated and nitrogen-containing functional groups capable of promoting hydrogen bonding, dipole interactions, and surface complexation [14,15,30].

Recent studies increasingly suggest that adsorption of pharmaceutical compounds may depend more strongly on surface chemistry and functional group distribution than on surface area alone [27,30]. Nevertheless, systematic comparisons between HTC-derived hydrochars and pyrolysis-derived biochars produced from the same biomass precursor under controlled conditions remain limited. In particular, there is insufficient experimental evidence directly correlating thermochemical conversion pathways, elemental composition, textural properties, and rapid adsorption performance for CBZ removal.

Therefore, the objective of this study was to valorize potato peel waste into carbonaceous adsorbents via hydrothermal carbonization and conventional pyrolysis and to systematically compare their physicochemical properties and adsorption performance toward carbamazepine removal from synthetic wastewater. The study further aimed to investigate the relationships between conversion pathways, surface chemistry, elemental composition, porosity, and adsorption behaviour. By evaluating whether low-severity HTC can outperform pyrolysis-derived biochars despite a lower BET surface area, this work contributes to the growing understanding of structure–property relationships in waste-derived adsorbents and highlights the potential of hydrothermal carbonization as a sustainable strategy for food-waste valorization and pharmaceutical remediation.

## 2. Results and Discussion

### 2.1. Sample Characterization

#### 2.1.1. BET Analysis

**Figure 1:**
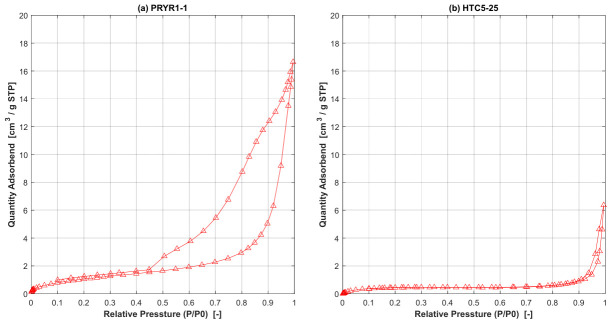
BET N_2_ isotherms of (**a**) the pyrolysis biochar (PRYR1-1) and (**b**) hydrochar from hydrothermal carbonisation (HTC5-25hr).

Figure 1a,b show the BET isotherm for the best performing biochars and hydrochars. The BET isotherms for the other samples are provided in the Supplementary Materials (Figures S1 and S2). The nitrogen adsorption–desorption isotherm of the potato peel biochar produced via pyrolysis exhibits the classical features of a Type IV mesoporous isotherm with an H3-type hysteresis loop, which is characteristic of non-rigid, slit-shaped pores commonly found in carbonaceous materials derived from lignocellulosic biomass [25]. The gradual increase in adsorbed nitrogen at low relative pressures (P/P_0_ < 0.1) suggests limited microporosity, while the more pronounced adsorption at intermediate-to-high relative pressures reflects extensive mesopore filling by capillary condensation.

The broad H3 hysteresis loop further suggests an abundance of open, plate-like pores and layered structures, consistent with pore formation through devolatilization during pyrolysis. Such mesoporous structures, combined with increased surface area, are commonly observed in biochars produced at elevated temperatures and are known to enhance intermolecular interactions and sorption accessibility [30].

In contrast, the potato peel hydrochar generated through HTC displays a Type II isotherm, which is typical of non-porous or weakly porous carbonaceous solids with dominant external surface adsorption [31]. The nearly horizontal adsorption plateau across mid-range relative pressures (P/P_0_ ≈ 0.1–0.9) suggests an absence of well-developed micro- or mesopores. The subtle hysteresis that emerges only at very high relative pressures (P/P_0_ → 1) corresponds mainly to condensation within macropores or interparticle voids rather than true internal pore networks. This behaviour aligns with the typically dense, microsphere-like morphology of hydrochars, which retain fewer structural voids and exhibit lower specific surface areas than pyrolyzed biochars due to the milder temperatures and water-mediated reactions involved in HTC.

The observed differences in isotherm behaviour indicate distinct textural characteristics between the pyrolysis-derived and HTC-derived materials. PRYR1-1 exhibited greater pore development and a higher measured BET surface area than HTC5-25hr, whereas HTC5-25h exhibited limited nitrogen-accessible porosity. Based on conventional adsorption expectations, materials with a greater surface area and mesoporosity are often expected to demonstrate enhanced adsorption performance.

However, the adsorption results obtained in this study did not follow this expected trend. Despite exhibiting a substantially lower BET surface area, HTC-derived materials showed higher apparent CBZ removal under the experimental conditions examined. This observation suggests that BET surface area alone was insufficient to explain adsorption behaviour in this system.

Because only N_2_-BET analysis, FTIR, CHNS analysis, and adsorption measurements were performed, the present data do not permit direct identification of the adsorption mechanism. The results therefore only indicate that factors other than measured surface area—including possible differences in surface accessibility, wettability, pore architecture, or surface chemistry—may contribute to the observed adsorption behaviour. Additional characterization (e.g., XPS, adsorption isotherms, zeta potential, calorimetry, or molecular modelling) would be required to establish mechanistic relationships.

Conversely, the hydrochar’s limited porosity and lower internal surface area constrain its total adsorption capacity, although its surface chemistry—typically richer in oxygen-containing functional groups—may facilitate specific interactions with certain antibiotic molecules, such as hydrogen bonding or electrostatic attraction. Such mechanisms have been observed in several hydrochar studies, where adsorption is consistent with surface functional groups rather than pore filling [32]. Nevertheless, due to structural limitations, hydrochars generally demonstrate slower diffusion rates and lower equilibrium capacities compared with corresponding biochars, unless chemically modified or activated. Overall, the biochar’s mesoporous structure makes it inherently more suited for the adsorption of bulky, aromatic antibiotics in wastewater, while hydrochar may require further activation or modification to achieve comparable performance [33,34,35].

Table 1 compares the BET surface areas and pore diameters of adsorbents produced from hydrothermal carbonization and pyrolysis of potato peel powders. Adsorbents with the prefix HTC were prepared via hydrothermal carbonisation, while those with the prefix PRYR were obtained through pyrolysis. Micropores, mesopores, and macropores are defined as pores with diameters <2 nm, 2–50 nm, and >50 nm, respectively [26]. Except for raw potato peel and HTC5-2hr, all adsorbents exhibited mesoporous structures, with pore diameters ranging from 2.60 nm to 19.77 nm.

Activated carbons typically exhibit higher surface areas than hydrochars [36]. This trend was observed for most samples; however, HTC5-25h (1.73 m^2^/g) surpassed PRYR2-1 (0.38 m^2^/g) and PRYR2-4 (1.52 m^2^/g), likely due to the influence of KOH dosage. Excessive KOH can damage pore walls through aggressive reactions with carbon, reducing surface area [31,37]. Consistent with the literature, HTC adsorbents generally had smaller pores than PRYR adsorbents [16].

Hydrothermal carbonization improved the surface area compared to raw potato peel, except for HTC5-2hr [10]. Longer residence times enhanced BET surface area, likely due to intensified hydrolysis and dehydration reactions at elevated temperatures, which release volatiles and create void spaces [15,30]. This trend was evident across HTC samples, where surface area increased with time. However, pore size did not consistently follow the expected increase with temperature and time; only HTC5-25hr exhibited a larger pore diameter than raw peel. The literature suggests that the liquid-to-solid ratio also influences surface area, with lower ratios causing incomplete carbonization [9]. HTC1-25hr and HTC5-25 aligned with this observation. Additionally, higher water ratios can promote macropore formation (>100 nm), but this was only partially supported by HTC5-25hr [12].

Pyrolysis generally produces activated carbons with high surface areas [7], and all PRYR samples exceeded raw peel in this regard. Pore diameters also increased post-pyrolysis, consistent with the literature [38]. Residence time influenced surface area variably: PRYR1 samples showed a slight decrease from 4.23 m^2^/g (1 h) to 3.77 m^2^/g (4 h), while PRYR2 samples increased from 0.38 m^2^/g to 1.52 m^2^/g. This discrepancy may reflect differences in pore development and collapse during prolonged activation [39,40]. Increasing the KOH ratio from 1 to 2 significantly reduced surface area across all PRYR samples, likely due to excessive carbon–KOH reactions damaging pore walls [31,37]. Contrary to expectations, pore diameters decreased with higher KOH ratios and longer residence times, suggesting structural collapse under aggressive activation [25,41]. However, all measured BET values are extremely low (<5 m^2^/g), far below most activated carbons. Importantly, all measured BET surface areas remained extremely low (<5 m^2^ g^−1^), and therefore, adsorption performance should not be interpreted as arising from extensive porosity development.

#### 2.1.2. Textural Analysis

**Figure 2:**
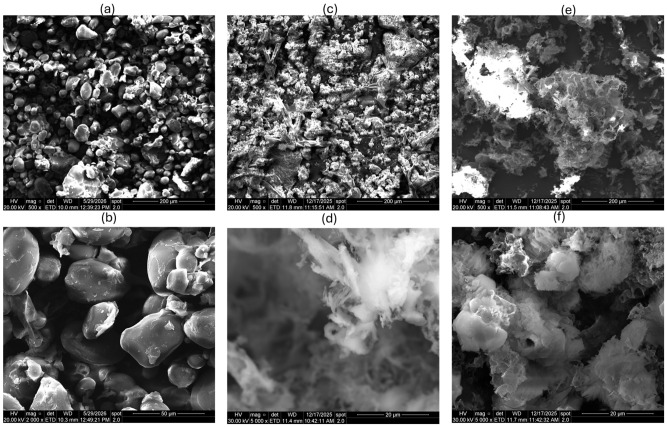
SEM images of (**a**,**b**) potato peel powder at different magnifications of ×500 and ×2000, respectively. (**c**,**d**) SEM images of PRYR1-1 adsorbent at magnifications of 500 and 5000, respectively. (**e**,**f**) HTC 5-25 at magnifications of 500 and 5000.

The SEM images in Figure 2a–f clearly illustrate the morphological evolution of potato peel powder as it undergoes pyrolysis and hydrothermal carbonisation. At magnifications of 500× and 2000×, the raw potato peel powder exhibits a fibrous, compact, and irregular surface, with limited porosity and visible remnants of collapsed plant cell structures. This morphology is consistent with previous reports showing that untreated potato peel retains its dense lignocellulosic matrix, characterized by layered tissues and minimal pore development [13]. Following pyrolysis, the PRY1-1 biochar displays a markedly different structure, with fractured surfaces, extensive fissures, and well-developed micropores, resulting from devolatilization and thermal degradation of organic components. Similar porous, brittle architectures have been reported for potato-waste-derived biochars, where volatile release during carbonisation generates interconnected pore networks and increases surface roughness [5,42]. In contrast, the HTC5-25 hydrochar exhibits a smoother, more spherical, and agglomerated morphology, with carbon microspheres and shallow surface pores visible at higher magnification. This morphology aligns with the well-established behaviour of hydrothermal carbonisation, where dehydration and polymerisation reactions promote the formation of spherical carbonaceous aggregates rather than the deep pore structures typical of pyrolysis [13].

Overall, SEM observations indicate clear morphological differences between the materials generated by pyrolysis and hydrothermal carbonisation. PRYR1-1 exhibited greater visible surface disruption and pore-like structures, whereas HTC5-25 appeared smoother and more aggregated.

However, SEM observations alone cannot quantify accessible surface area or establish adsorption mechanisms. Although morphology may influence adsorption behaviour, the present images do not explain the superior adsorption observed for HTC materials. The SEM results are therefore interpreted only as qualitative evidence of structural differences rather than proof of adsorption pathways. The comparative interpretation of the textural properties of the three different materials is summarized in Table 2.

#### 2.1.3. Elemental Analysis (CHNS)

CHNS analysis provides the composition of carbon, hydrogen, nitrogen, and sulphur in an adsorbent. Table 3 below shows the CHNS results for all eight adsorbents and raw potato peel.

The elemental composition of the raw potato peel (PP-Raw) was 39.77% C, 6.38% H, and 2.09% N, in close agreement with reported values for potato peel wastes (≈41.9% C, 5.6% H, 1.6% N), confirming that the feedstock used here is representative of the literature baselines [8]. For the HTC hydrochars, carbon increased with residence time in both water-to-biomass series (HTC1: 54.23→62.17% C; HTC5: 37.09→53.28% C), consistent with the expectation that prolonged HTC promotes dehydration, decarboxylation, and aromatization, thereby enriching fixed carbon and lowering oxygen [13,14]. Lower water loading (i.e., higher solid-to-liquid ratio; HTC1 vs. HTC5 at the same time) also yielded higher carbon contents, aligning with reports that increased solids concentration can enhance carbonization, albeit with potential trade-offs in mixing and heat transfer [2]. Hydrogen typically declines with HTC severity due to dehydration and cleavage of C–H/O–H bonds [43,44]. This trend held for HTC5 (6.80→5.24% H), but a slight rise for HTC1 (5.27→5.74% H) suggests subtle structural differences; the observed intensification of C–H stretching near ~2900 cm^−1^ for the longer residence sample is a plausible indicator of residual aliphatic functionality that would elevate measured hydrogen. Nitrogen generally increased with residence time in both ratio series (HTC1: 2.44→2.85% N; HTC5: 1.25→2.55% N), in line with prior reports of N enrichment via condensation/polymerization under HTC conditions [16]. In contrast to studies that show higher liquid-to-solid ratios elevating N retention in hydrochars, the present data display the opposite, which can be rationalized by increased dissolution of nitrogenous species into the aqueous phase at higher water loadings [45,46].

For the pyrolysis (PRYR) chars, the literature commonly reports that increasing residence time (or severity) raises fixed carbon and reduces hydrogen as volatiles are expelled and aromaticity grows [47]. Here, however, carbon decreased with time in both series (PRYR1: 16.59→13.95% C; PRYR2: 12.36→10.02% C). Hydrogen followed the expected decline only in PRYR1 (1.10→0.66% H) but increased in PRYR2 (1.13→1.45% H).

These deviations from commonly reported pyrolysis trends suggest that the materials produced under the present conditions behaved differently from typical high-severity pyrolysis systems reported in the literature.

Several factors—including process severity, ash accumulation, mineral concentration effects, analytical uncertainty, or incomplete conversion—could potentially contribute to these observations. However, because complementary analyses such as ash quantification, TGA, repeat elemental measurements, or mineral analysis were not performed, these explanations remain speculative.

Accordingly, CHNS data are interpreted here as descriptive indicators of compositional variation rather than mechanistic evidence linking elemental composition directly to adsorption performance [2,43,44,45,46,47].

#### 2.1.4. FTIR Analysis

**Figure 3:**
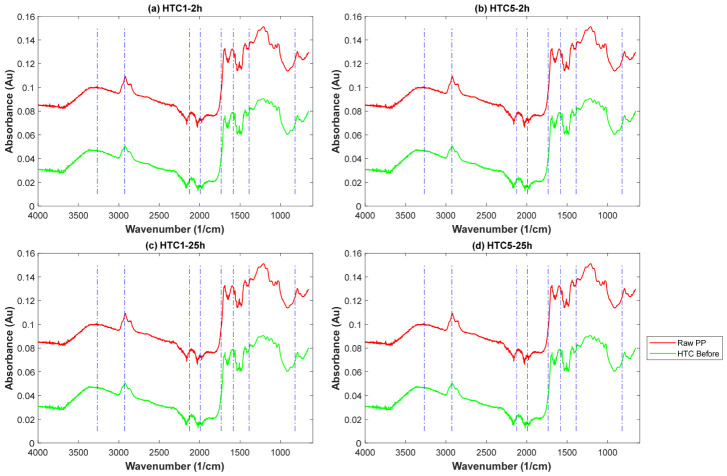
FTIR results of raw potato peel and the HTC adsorbents before adsorption ((**a**) HTC1-2h, (**b**) HTC5-2h, (**c**) HTC1-25h, (**d**) HTC5-25h). The dashed blue lined show important functional groups.

FTIR spectroscopy was employed to identify functional groups present in raw potato peel and its derived adsorbents prepared via HTC and pyrolysis (PRYR). These functional groups are critical in understanding adsorption mechanisms, particularly for organic pollutants such as carbamazepine (CBZ), which is a neutral, hydrophobic pharmaceutical compound. Figure 3 shows the comparison between the FTIR spectrum of raw potato and that of HTC samples.

The FTIR spectrum of raw potato peel exhibited a broad band between 3000 and 3300 cm^−1^, attributed to hydrogen-bonded –OH stretching vibrations of hydroxyl groups, indicating the presence of cellulose and hemicellulose components [48].

A peak at 2938 cm^−1^ corresponds to aliphatic C–H stretching of methyl and methylene groups in polysaccharides [49]. The band at 1632 cm^−1^ is associated with C=O stretching vibrations of aldehydes, suggesting lignin-derived aromatic structures [50,51]. Peaks between 1000 and 1350 cm^−1^ are linked to C–O and C–C stretching in starch molecules [52], while absorptions at 1600–1400 cm^−1^ indicate aromatic C=C vibrations [6]. These oxygen-containing groups may influence hydrophilicity and may provide sites capable of interacting with aqueous contaminants, although FTIR alone cannot confirm adsorption mechanisms.

HTC-derived adsorbents displayed characteristic peaks at 3753 cm^−1^ (–OH stretching), 2920–2850 cm^−1^ (C–H stretching), and 1693 cm^−1^ (C=O stretching of aromatic skeleton), confirming the presence of hydroxyl, carbonyl, and aromatic functionalities [13]. Peaks around 1570 cm^−1^ correspond to aromatic C=C vibrations, while bands between 1195 and 594 cm^−1^ indicate substituted –CH groups in benzene rings. Hydrothermal treatment appeared to retain oxygen-containing functionalities relative to pyrolysis-derived materials. These functionalities may contribute to adsorption behaviour; however, no direct evidence was collected to confirm their specific role in CBZ uptake [53].

**Figure 4:**
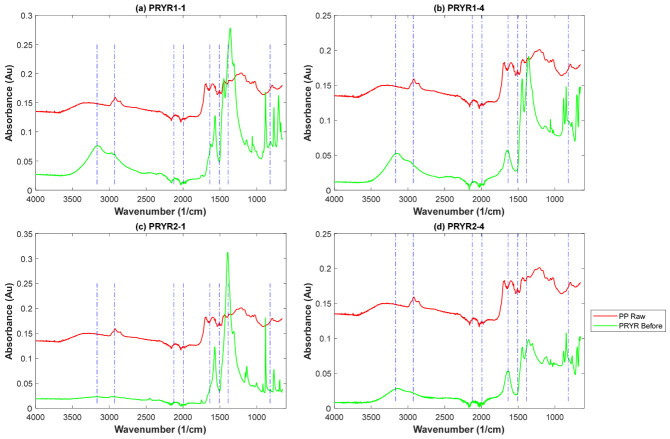
FTIR results of raw potato peel and the PRYR adsorbents before adsorption ((**a**) PRYR1-1, (**b**) PRYR1-4, (**c**) PRYR2-1, (**d**) PRYR2-4)). The dashed blue lined show important functional groups.

In Figure 4, pyrolysis-derived adsorbents exhibited broad O–H stretching bands (3100–3400 cm^−1^), aromatic C=C stretching at 1615 cm^−1^, and C–OH related vibrations at 1050–1150 cm^−1^ [53,54]. Pyrolysis generally increases aromaticity and surface hydrophobicity, favouring π–π electron donor–acceptor interactions with CBZ’s aromatic rings. However, the reduction in polar oxygenated groups may limit hydrogen bonding compared to HTC adsorbents.

The observed functional groups provide indirect information regarding potential adsorbent–adsorbate interactions. Based on the literature precedent, hydrogen bonding, π–π interactions, hydrophobic partitioning, and pore accessibility represent plausible adsorption pathways. However, the present FTIR data do not allow the relative contribution of these mechanisms to be quantified. HTC adsorbents, rich in oxygenated groups, may exhibit higher affinity for CBZ through combined hydrogen bonding and hydrophobic interactions. Conversely, PRYR adsorbents, with enhanced aromaticity and porosity, favour π–π interactions and van der Waals forces, which are crucial for adsorbing neutral hydrophobic pharmaceuticals [53,54,55].

**Figure 5:**
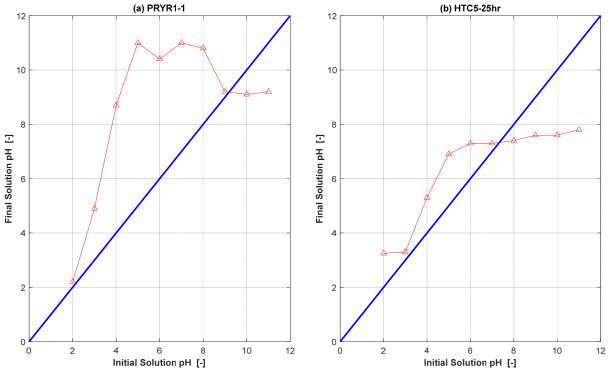
Point of zero charge results for the two adsorbent samples (**a**) PRYR1-1 and (**b**) HTC5-25. The blue line is line of y = x.

The point of zero charge ($pH_{PZC}$) results in Figure 5 indicate that the KOH-activated pyrolysis biochar (PRYR1-1) possesses a higher $pH_{PZC}$ (≈9.1–9.3) than the hydrothermal carbonized material HTC5-25 (≈7.2–7.4). At pH values below the $pH_{PZC}$, the adsorbent surface is positively charged, whereas above the $pH_{PZC},$ the surface becomes negatively charged. The higher $pH_{PZC}$ of PRYR1-1 suggests that NaOH activation and pyrolysis generated a more alkaline surface enriched with basic oxygen-containing groups, mineral ash, and aromatic carbon domains. In contrast, HTC5-25hr exhibits a moderately neutral surface chemistry typical of hydrochars produced at lower temperatures, where oxygenated functionalities such as hydroxyl, carbonyl, and carboxyl groups remain abundant. Similar trends have been reported for chemically activated biochars, where alkaline activation increases surface basicity and aromaticity while hydrothermal carbonization preserves polar functional groups [29].

These surface charge characteristics have important implications for carbamazepine (CBZ) adsorption. CBZ is largely neutral within the environmentally relevant pH range (pKa ≈ 13.9), meaning electrostatic attraction is not the dominant adsorption mechanism. Because CBZ remains largely neutral under the tested conditions, electrostatic interactions are expected to be less dominant than for ionisable contaminants. Nevertheless, the present experiments do not allow direct determination of which interactions controlled adsorption. Consequently, both adsorbents are expected to remove CBZ effectively over a broad pH range despite differences in $pH_{PZC}$. However, the highly alkaline PRYR1-1 surface may enhance π–π interactions because pyrolysis and NaOH activation increase graphitic and aromatic domains, which favour adsorption of aromatic pharmaceuticals such as CBZ. NaOH activation is also known to generate additional microporosity and oxygen-containing groups that improve CBZ uptake [29].

The HTC5-25hr hydrochar, despite its lower $pH_{PZC}$, may still exhibit strong CBZ adsorption because hydrothermal carbonization retains abundant surface functional groups capable of hydrogen bonding with the amide group of CBZ. Hydrochars generally contain fewer well-developed pores than pyrolysis biochars, but their oxygen-rich surfaces can compensate through specific interactions with pharmaceutical molecules. Recent studies on biomass-derived hydrochars and activated biochars similarly showed that CBZ adsorption is strongly influenced by surface chemistry rather than surface charge alone [56].

Compared with the literature values, the observed $pH_{PZC}$ range is consistent with other activated carbons and biochars used for CBZ removal. KOH-activated pomelo peel biochar reported by Chen et al. exhibited strong CBZ adsorption capacities up to 286.5 mg g^−1^, attributed mainly to π–π interactions and pore structure development. Likewise, NaOH-activated algae biochar achieved CBZ capacities above 118 mg g^−1^, with hydrogen bonding identified as a major adsorption mechanism [57]. Overall, $pH_{PZC}$ measurements indicate differences in surface characteristics between the two adsorbents but should not be interpreted as direct evidence of adsorption mechanisms.

### 2.2. Adsorption Results

**Figure 6:**
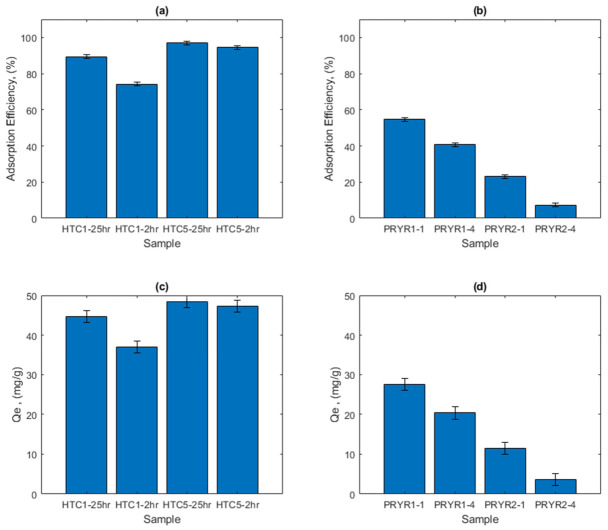
(**a**,**b**) Adsorption efficiencies for CBZ uptake of (**a**) hydrothermal carbonisation of hydrochar and (**b**) pyrolysis of potato peel biochars. Uptake of CBZ (**c**) pyrolysis of potato hydrochars and (**d**) pyrolysis of potato peel biochars. Conditions: CBZ initial concentration (50 mg/L); adsorbent dosage (1 g/L); solution pH 6, room temperature (~20 °C); static condition (no agitation); contact time, 1 min. Experiments were performed in duplicates.

The adsorption performance of HTC hydrochars and PRYR biochars for carbamazepine (CBZ) removal after 1 min of contact with a 50 mg/L CBZ solution (1 g/L dosage) reveals notable differences in removal efficiency and uptake. The results are presented in Figure 6.

**Figure 7:**
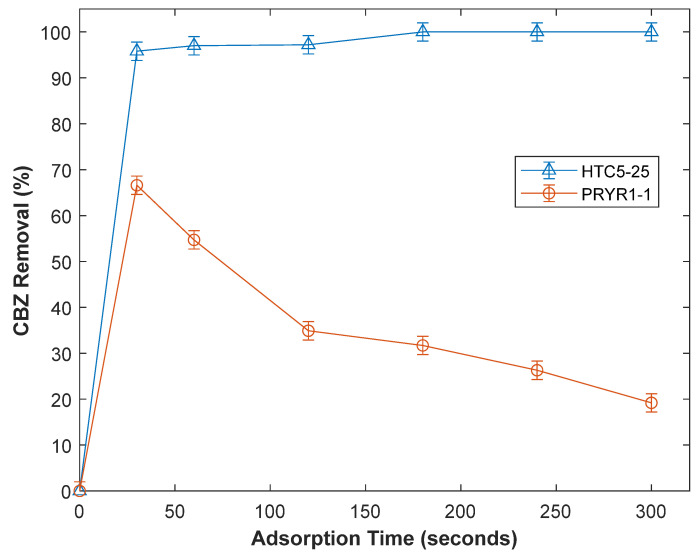
Comparison of the adsorption kinetics of HTC and PRYR samples in the first 5 min of adsorption. Conditions: CBZ initial concentration (50 ppm); adsorbent dosage (1 g/L); solution pH 6, room temperature (~20 °C); static condition (no agitation). All experiments were performed in duplicates.

HTC-derived hydrochars demonstrate outstanding adsorption efficiency, achieving approximately 12–100% removal (Figure 7a) and high uptake values between 35 and 50 mg/g (Figure 7c). These capacities are comparable to or exceed those reported for activated hydrochars in the literature. For instance, [28] prepared a dual-activator modified hydrochar with a CBZ uptake of ~296 mg/g, attributed to its high specific surface area and π–π and pore-filling interactions. Although the technique in this study used less aggressive activation, the observed uptakes still reflect the benefits of hydrothermal processing [28] [22].

By contrast, PRYR biochars show much lower performance, with removal efficiencies of ~10–40% and Qₑ values between 5 and 20 mg/g (Figure 7b,d). These results suggest biochars produced via pyrolysis under mild conditions retain fewer oxygenated surface groups, leading to weaker interactions with CBZ molecules. This aligns with findings from [19], who reported typical CBZ adsorption capacities on biochars and activated carbons ranging from 10 to 40 mg/g, depending heavily on precursor and pyrolysis/activation conditions.

The observed performance difference indicates that adsorption behaviour under the tested conditions did not scale directly with the BET surface area. Although this observation is consistent with the literature, suggesting that surface chemistry can influence pharmaceutical adsorption, the present dataset does not establish causality. Hydrothermal carbonization preserves and introduces polar oxygen-functional groups and moderate porosity, which promote CBZ adsorption through hydrogen bonding, π–π interactions, and pore filling. Similar mechanisms were emphasized by [20] in their study on magnetic biochar, where pore-filling and π–π interactions drove adsorption.

Under the specific experimental conditions investigated (50 mg/L CBZ, pH 6, 1 g/L dosage, synthetic water, short contact time), HTC-derived materials exhibited higher apparent removal efficiency and uptake than the corresponding pyrolysis-derived materials. These results should be interpreted as comparative screening data rather than comprehensive adsorption performance metrics.

Statistical Analysis

The influence of process parameters on carbamazepine (CBZ) removal was evaluated using one-way ANOVA for both pyrolysis and hydrothermal carbonisation systems. The results are summarized in Tables S1 and S2.

The ANOVA results revealed that both the KOH:biomass mass ratio (x_1_) and pyrolysis time (x_2_) exerted statistically significant effects on CBZ removal efficiency (*p* < 0.05). The exceptionally high F-values (1466.44 and 350.22, respectively) indicate strong sensitivity of CBZ adsorption to these parameters. The minimal residual error (Sum Sq = 0.72) confirms excellent model fit and low experimental variability. These findings suggest that CBZ removal primarily reflects activation intensity and residence time, which together enhance surface area, pore accessibility, and aromaticity of the resulting biochar.

In contrast, the water: biomass mass ratio (x_1_) and reaction time (x_2_) showed no statistically significant influence (*p* > 0.05) on CBZ removal. The relatively high error term (Sum Sq = 989.1) indicates greater variability and weaker dependence on process control. This outcome indicates that within the limited experimental space investigated, no statistically detectable HTC process effect was observed. Broader experimental conditions may produce different outcomes.

#### 2.2.1. Adsorption Kinetics

The adsorption kinetics shown in Figure 7 reveal a substantial difference between the HTC hydrochar and pyrolysis-derived biochar. HTC5-25hr achieved approximately 95% CBZ removal within the first 30 s and approached complete removal thereafter. In contrast, PRYR1-1 achieved a maximum removal of approximately 65% before gradually declining to around 20% after 300 s.

The extremely rapid uptake observed for HTC5-25hr suggests the presence of readily accessible adsorption sites and favourable adsorbate–surface interactions. Such behaviour is characteristic of systems in which external surface adsorption and adsorption onto easily accessible mesoporous structures dominate the initial uptake stage. Similar rapid adsorption phenomena have been reported for hydrochars and activated carbons used for pharmaceutical removal, where oxygen-containing functional groups contribute significantly to adsorption affinity [22,28].

The decline in removal observed for PRYR1-1 may indicate weaker adsorption interactions and partial desorption of previously adsorbed CBZ molecules. The FTIR and elemental analysis results suggest that the pyrolysis-derived materials possessed fewer oxygen-containing functional groups capable of forming strong hydrogen-bonding interactions with CBZ. Consequently, adsorption was likely dominated by weaker hydrophobic and π–π interactions. Similar behaviour has been reported for CBZ adsorption on biochars, where adsorption strength was strongly influenced by carbon structure and mineral composition [58].

Comparison with the literature indicates that the rapid adsorption achieved by HTC5-25hr is highly competitive. While many activated carbons and engineered biochars require contact times ranging from tens of minutes to several hours to achieve equilibrium, the present HTC hydrochar attained near-equilibrium adsorption within seconds. This rapid adsorption behaviour is advantageous for practical water treatment applications because it reduces reactor volume requirements and improves process efficiency.

The results collectively indicate that adsorption kinetics are governed not only by the pore structure but also by the availability of functional groups capable of forming strong interactions with CBZ molecules. The HTC route therefore appears particularly promising for developing fast-acting adsorbents for pharmaceutical remediation.

#### 2.2.2. Proposed Interpretation of Adsorption Behaviour

##### Effect of Physicochemical Properties

Based on the provided data presented in Table 4, the correlation between CBZ removal efficiency and the physicochemical properties of HTC hydrochars and PRYR biochars reveals a complex interplay of factors, rather than a dependency on any single parameter.

Firstly, examining the elemental composition and atomic ratios suggests that the degree of carbonization and surface chemistry may contribute to adsorption behaviour. For the HTC series, the highest CBZ removals (94.4% for HTC1-25hr and 97.0% for HTC5-25hr) correspond to higher carbon content (C%) and lower H/C atomic ratios (1.11 and 1.18, respectively). A lower H/C ratio typically suggests higher aromaticity and carbonization [59], which can enhance π-π electron donor–acceptor (EDA) interactions with the aromatic rings of CBZ [58]. Conversely, the PRYR biochars, despite sometimes having higher sulphur content (e.g., PRYR1-1, PRYR1-4), show markedly lower CBZ removal (23.0% to 54.7%). Their lower carbon content and varying H/C ratios suggest a less graphitized, potentially more oxidized structure, which may be less favourable for π-π interactions with CBZ.

Regarding physical properties, the BET surface area shows a notable but non-linear relationship with adsorption. The HTC samples with the highest removal (HTC5-25hr, 97.0%) possess a moderate BET area (1.733 m^2^/g), which is substantially lower than that of PRYR1-1 (4.226 m^2^/g), yet the latter achieves only 54.7% removal. This suggests that while the surface area provides potential adsorption sites, it is not the sole determinant of efficiency for CBZ. More critically, the pore diameter appears to play a significant role in accessibility. The HTC samples with high removal have pore diameters between 2.60 and 5.06 nm (mesoporous range), which may be optimal for accommodating the CBZ molecule (kinetic diameter ~0.7 nm) and facilitating diffusion [60]. In contrast, PRYR samples with very large pore diameters (e.g., 19.77 nm for PRYR1-1) may represent more macroporous structures with fewer adsorption sites per volume, potentially explaining their lower performance despite higher surface area. Notably, the raw material (PP-Raw) with minimal surface area and inconclusive data for some HTC samples underscores that the development of a porous structure via hydrothermal carbonization is crucial for creating effective adsorbents.

##### FTIR Observations Before and After Adsorption

**Figure 8:**
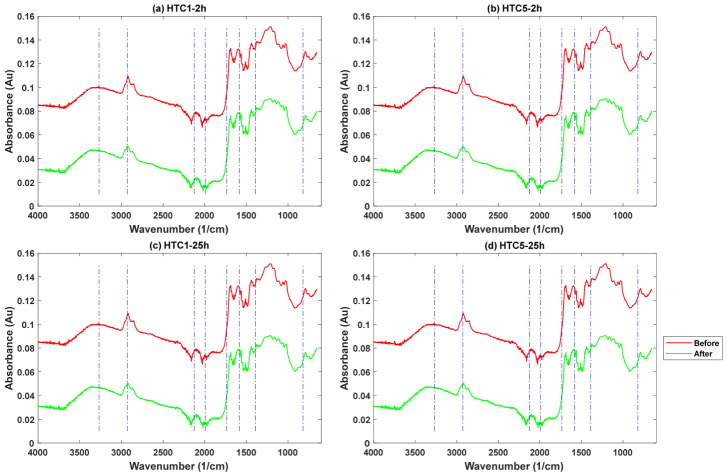
Comparison of the FTIR spectra of the HTC hydrochars before and after adsorption. The dashed blue lined show important functional groups.

**Figure 9:**
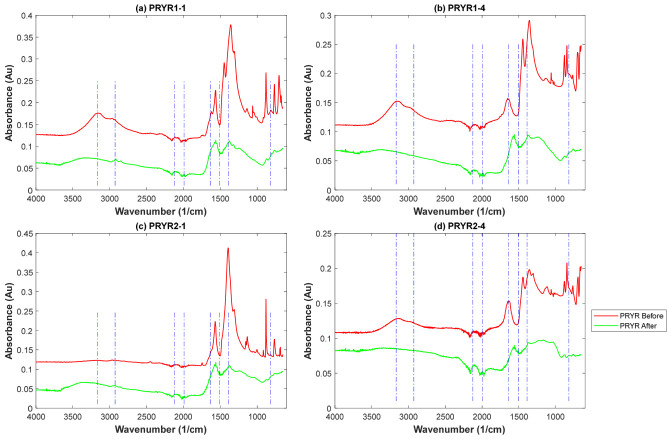
Comparison of the FTIR spectra of the PRYR biochars before and after adsorption. The dashed blue lined show important functional groups.

The FTIR spectra (Figure 8 and Figure 9) provide insight into the adsorption mechanisms. For HTC adsorbents (Figure 8), the attenuation or shift in bands in regions associated with O-H stretching (~3400 cm^−1^) and C=O/C-O groups (~1700–1000 cm^−1^) after adsorption suggests the involvement of these functional groups in CBZ uptake, likely through hydrogen bonding or dipole–dipole interactions [61].

For PRYR biochars (Figure 9), changes in similar spectral regions are also observable. The presence of sulphur-containing groups in some PRYR samples (indicated by elemental analysis) could also participate in specific interactions, though their overall lower performance suggests that these are less effective for CBZ than the combined π-π and polar interactions facilitated by the HTC chars. The consistent pattern across both figure sets suggests that CBZ adsorption involves surface functional groups, complementing the likely dominant mechanism of π-π conjugation between the adsorbent’s aromatic backbone and the CBZ molecule [58,62].

The observed spectral changes are consistent with possible involvement of surface functional groups during adsorption. However, FTIR alone cannot establish mechanisms or quantify contributions from individual interactions.

##### Comparison with the Literature and Proposed Interpretation

Table 5 compares the CBZ adsorption performance of HTC5-25hr and PRYR1-1 with several adsorbents reported in the literature. Among the materials studied, HTC5-25hr exhibited a notably high adsorption capacity of 47.5 mg/g despite having a very low surface area of only 1.73 m^2^/g. When normalized to surface area, HTC5-25hr achieved the highest adsorption efficiency (28.03 mg/m^2^), indicating that its adsorption performance is not primarily a reflection of surface area, but rather by the presence of effective surface functional groups and favourable surface chemistry generated during hydrothermal carbonisation of potato peel. In addition, HTC5-25hr reached adsorption equilibrium within only 1 min, demonstrating extremely rapid adsorption kinetics compared to most literature-reported adsorbents, which generally require several hours.

PRYR1-1 also showed strong performance, with an adsorption capacity of 27.4 mg/g and a normalized capacity of 6.32 mg/m^2^, again achieved within 1 min. Although its overall adsorption capacity was lower than HTC5-25hr, it still outperformed several conventional carbonaceous adsorbents in terms of adsorption efficiency relative to surface area and contact time. The rapid adsorption observed for both potato peel-derived materials suggests that accessible active sites and surface interactions may play a more important role than extensive porosity alone.

In comparison, adsorbents such as RH-AC, ZCA-CSC, BAHC, and MH-HTC-800-1:1 exhibited much larger surface areas (906–1729 m^2^/g) and higher absolute adsorption capacities (190–384 mg/g). However, their normalized adsorption capacities were substantially lower (0.187–0.297 mg/m^2^), and adsorption required much longer contact times, typically 24 h or more. Similarly, BL700 and MTPEE showed comparatively poor adsorption capacities and efficiencies despite moderate surface areas. Overall, the results demonstrate that HTC5-25hr and PRYR1-1 are highly efficient low-surface-area adsorbents for CBZ removal, with exceptional adsorption rates and superior surface-area-normalized performance compared to many previously reported materials.

**Figure 10:**
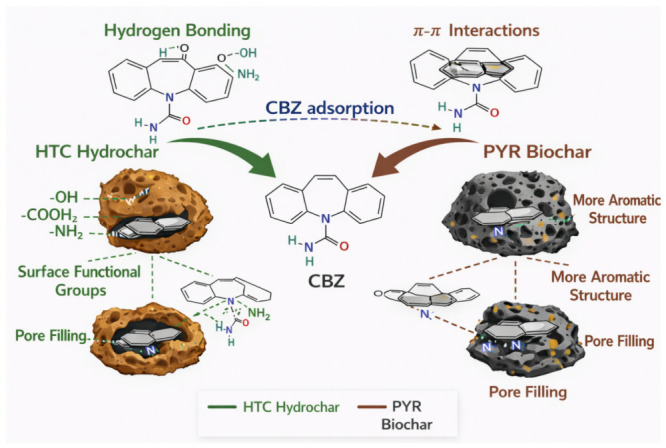
Proposed CBZ removal mechanism by HTC and PRYR adsorbents.

In contrast, PYR biochar exhibits a more carbonized, aromatic, and hydrophobic surface due to high-temperature treatment, leading to different dominant adsorption mechanisms. π–π interactions between the aromatic rings of CBZ and the graphitic domains of the biochar are a key pathway for removal (Figure 10). Hydrophobic interactions further enhance adsorption, as CBZ preferentially partitions from the aqueous phase onto the nonpolar biochar surface. The well-developed microporosity and higher surface area of PYR biochar also promote pore filling, increasing adsorption capacity through physical entrapment. Additionally, residual mineral components in the biochar, such as metal cations (e.g., Na^+^, Fe^2+^, Zn^2+^), can contribute to adsorption via cation bridging or coordination with CBZ functional groups [66,67,68,69].

Overall, the schematic emphasizes that HTC hydrochar relies more on hydrogen bonding and polar interactions, whereas PYR biochar removal is dominated by π–π interactions, hydrophobic effects, and enhanced pore filling, reflecting the differences in surface chemistry and structure resulting from their respective production processes [33,68].

The results suggest that CBZ adsorption may reflect a combination of hydrogen bonding, π–π electron donor–acceptor interactions, hydrophobic interactions, and pore-filling mechanisms. Although PRYR1-1 possessed a larger BET surface area (4.23 m^2^ g^−1^) than HTC5-25hr (1.73 m^2^ g^−1^), the HTC material achieved almost twice the CBZ removal efficiency. This suggests that surface chemistry and pore accessibility were more important than total surface area alone. Similar observations have been reported by [58], who demonstrated that CBZ adsorption is strongly influenced by carbon structure and surface functional groups rather than solely by surface area. Likewise, Ref. [19] concluded that oxygen-containing functionalities and aromatic domains play critical roles in CBZ adsorption on biochars and activated carbons.

The FTIR results support this interpretation, as HTC materials retained abundant hydroxyl and carbonyl functionalities capable of forming hydrogen bonds with the amide group of CBZ. Combined with mesoporous structures that facilitate rapid diffusion, these functionalities likely contributed to the exceptionally rapid adsorption observed for HTC5-25hr. Similar mechanisms have been reported for hydrochars and activated carbons prepared from biomass feedstocks [28,56].

Overall, the present results suggest that adsorption behaviour under the investigated conditions may depend on multiple interacting factors rather than a single measurable property. Although HTC materials demonstrated higher apparent CBZ removal than PRYR materials, the current dataset is insufficient to establish definitive adsorption mechanisms.

Future studies employing equilibrium isotherms, independent analytical confirmation, spectroscopic analysis, and broader operating conditions are required before mechanistic conclusions can be drawn.

## 3. Materials and Methods

### 3.1. Preparation of the Adsorbents

**Figure 11:**
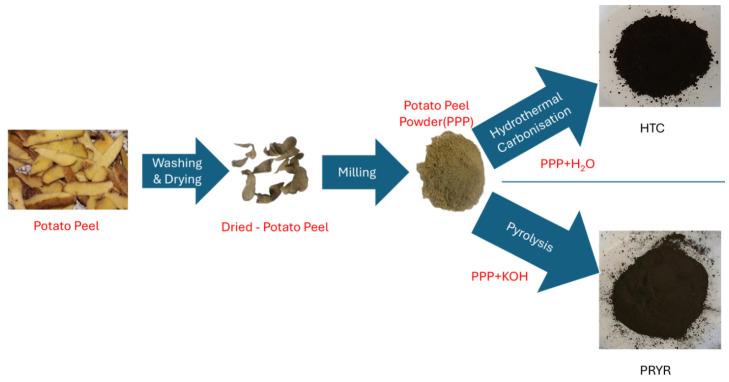
Production scheme for HTC and PRYR adsorbents.

The production scheme of the adsorbents is presented in Figure 11. Samples were produced from dried potato peel powder using potassium hydroxide, KOH (purchase from Merck, Billingham UK) as an activation agent, under different conditions of reaction times and KOH ratios, whilst keeping the reaction temperature at 400 °C. The pyrolysis reaction time was either 1 or 4 h, whilst the KOH/biomass ratio was either 1:1 or 2:1. In total, 10 g of potato peel was used in this case, and the mass of KOH used was 10 g for the ratio 1:1 or 20 g for the ratio 2:1. The samples produced by pyrolysis are named using the convention PRYRX-Y, where X represents the parts of KOH by mass and Y represents the pyrolysis reaction time. For instance, a sample produced with a KOH: biomass ratio of 2:1 and a reaction time of 1 h would be named PRYR2-1.

Hydrochar was produced in an autoclave reactor vessel from dried potato peel powder with distilled water. During the hydrothermal carbonisation, the reaction temperature was kept constant at 200 °C while varying the reaction time (2 or 25 h) and weight/volume ratios of the biomass to distilled water (1:1 or 1:5). For the ratio of 1:1, 4 g of potato peel and 4 mL of distilled water were used. In total, 10 g of potato peel and 50 mL of distilled water were used for the ratio of 1:5. The samples produced by hydrothermal carbonisation are named using the convention HTCX-Yhr, where X represents the parts of distilled water by mass/volume and Y represents residence time. For instance, a sample produced with a biomass: distilled water ratio of 1:1 and a residence time of 2 h would be named HTC1-2h.

### 3.2. Characterization of the Samples

The functional groups of the adsorbents PPPYR and PPHTC (both before and after adsorption) were identified using Fourier-transform infrared (FTIR) spectroscopy within the range of 4000–400 cm^−1^ [24]. CHNS analysis was also conducted in a Perkin Elmer CHNS analyzer PE2400CHNS (Perkin Elmer, Springfield, USA) using a procedure previously described [70]. The surface area and porosity information of each adsorbent (before adsorption) were evaluated using Brunauer–Emmett–Teller (BET) analysis at 77 K using a Micromeritics Tristar 3020 instrument (Malvern Panalytical, Worcestershire, UK) with N_2_ gas flow [24]. For scanning electron microscopy (SEM) analysis, the samples were coated with gold for electron reflection prior to analysis using a JOEL JSM-6400 microscope and JSM-7900F (Joel Ltd., Tokyo, Japan) using an acceleration voltage of 20 keV [71].

The point of zero charge of the HTC5-25hr and PRYR1-1 sample was determined using the method previously described in [72]. Briefly, a series of solutions was prepared with 50 mg of adsorbent in 50 mL of deionized water with pH in the range 2 to 11. The pH values were adjusted using 0.1 M solutions of HCl and NaOH. The samples were then agitated at 100 rpm for 24 h at room temperature. The final pH values were then measured and plotted against the initial pH values. The pH of the point of zero charge was determined as the point of intersection of the final pH curve line with the line y = x.

### 3.3. Adsorption Study

A 50 ppm aqueous solution of CBZ was produced from a 100 ppm stock solution by dilution, and the pH was adjusted to 6 using HCl and NaOH. In total, 10 mL of the CBZ solution was transferred into 4–20 mL glass vials, followed by the addition of 10 mg of each of the adsorbents. At the expiry of the required set contact time, the concentration of the CBZ solutions was measured using a UV-VIS spectrophotometer at a wavelength of 285 nm. The measured absorbance from UV-VIS spectrophotometry was used in conjunction with calibration data to determine the concentration of CBZ in solution samples. The calibration data is provided in Supplementary Materials Figure S3.

The removal of efficiency of the adsorbent was determined from the following equation:

(1) $$PR=100\left(\frac{C_{o}-C_{e}}{C_{o}}\right)\;\%$$

In the equation, $C_{o}$ and $C_{e}$ are the initial and final concentrations of the CBZ before and after adsorption.

The CBZ uptake by the adsorbent, $q_{e}$ (mg/g), was determined from

(2) $$q_{e}=V\left(\frac{C_{o}-C_{e}}{m}\right)$$

where V is the volume of the CBZ solution, m is the mass of the adsorbent used in the test, and $C_{o}$ and $C_{e}$ are as defined previously.

### 3.4. Statistical Analysis

To investigate the influence of process variables such as solids loading (water: biomass mass ratio), reaction time on the CBZ removal performance of the HTC adsorbent, a 2-level factorial design, and corresponding ANOVA analysis were conducted using a custom-written MATLAB script (Version 2024a). For the PRYR adsorbent, the process variables were KOH: biomass ratio and pyrolysis time.

## 4. Conclusions

This study compared hydrothermal carbonization (HTC) and pyrolysis for the production of potato-peel-derived adsorbents for carbamazepine (CBZ) removal from water. Among the materials investigated, HTC5-25hr exhibited the highest performance, achieving approximately 97% CBZ removal and rapid adsorption within the first few minutes of contact.

The superior performance of the HTC-derived adsorbents may be attributed primarily to their surface chemistry, including oxygen-containing functional groups and favourable pore structures that promoted hydrogen bonding, π–π interactions, hydrophobic interactions, and pore filling. Statistical analysis showed that pyrolysis conditions significantly influenced adsorption performance, whereas the hydrothermal carbonization variables investigated did not produce statistically significant differences within the experimental range studied.

Although HTC5-25hr demonstrated the highest CBZ removal efficiency, its production required a relatively long processing time. Therefore, the selection of an optimal adsorbent should consider not only adsorption performance but also energy requirements and overall process sustainability. Future studies should evaluate these factors through detailed techno-economic and life-cycle assessments.

Overall, the results demonstrate the potential of potato-peel-derived hydrochars as effective adsorbents for pharmaceutical removal and highlight the importance of surface chemistry in governing adsorption performance.

### Recommendations for Future Work

Future studies should investigate the influence of hydrothermal carbonization temperature, residence time, and water-to-biomass ratio over a broader operating range to further optimize hydrochar properties and adsorption performance. Emphasis should be placed on evaluating adsorbent regeneration, reusability, and long-term stability to determine the practical feasibility of large-scale implementation.

A major priority for future work should be the development of comprehensive adsorption isotherm models. Equilibrium studies conducted over a wide concentration range would enable the application of Langmuir, Freundlich, Sips, Temkin, and Dubinin–Radushkevich isotherms to better understand adsorption capacity, surface heterogeneity, and adsorption energetics. Such modelling would provide important mechanistic insight and allow direct comparison with other adsorbents reported in the literature.

Similarly, detailed adsorption kinetic modelling should be undertaken to quantify the rate-controlling mechanisms governing CBZ uptake. Application of pseudo-first-order, pseudo-second-order, Elovich, intraparticle diffusion, and Boyd models would help distinguish between external mass transfer, pore diffusion, and surface reaction processes. Given the exceptionally rapid uptake observed for HTC5-25hr, kinetic modelling is particularly important for identifying the mechanisms responsible for the near-instantaneous adsorption behaviour.

Future investigations should also include thermodynamic analyses to determine the spontaneity, enthalpy, and entropy changes associated with adsorption, thereby providing a deeper understanding of adsorbate–adsorbent interactions. Testing in real wastewater matrices containing dissolved organic matter, competing ions, and multiple pharmaceutical contaminants is also required to assess performance under realistic operating conditions.

Finally, continuous-flow column studies, process-scale modelling, life-cycle assessment, and techno-economic analysis should be performed to evaluate the scalability and commercial potential of potato-peel-derived hydrochars for sustainable pharmaceutical removal from water.

## Figures and Tables

**Table 1:** Comparison between the BET surface areas and pore diameters of the adsorbent produced from different processes.

Adsorbent	BET Surface Area (m^2^/g)	Pore Diameter (nm)
PP-Raw	0.12± 0.01	5.05
HTC1-2h	0.73 ± 0.03	3.65
HTC1-25h	0.95 ± 0.01	2.60
HTC5-2h	Inconclusive	-
HTC5-25h	1.73 ± 0.04	5.06
PRYR1-1	4.23 ± 0.03	19.77
PRYR1-4	3.77 ± 0.03	14.15
PRYR2-1	0.38 ± 0.01	11.74
PRYR2-4	1.52 ± 0.01	6.25

**Table 2:** Comparison of textural properties of the adsorbent based on SEM images.

Material	Macro-Scale Features	Micro-Scale Features	Potential Implications for Adsorption (Hypothesized)
Raw potato peel powder	Fibrous, irregular, dense	Limited porosity, shallow cracks	Low surface area; limited adsorption sites
PRYR1-1 (pyrolysis)	Brittle, fractured, porous	Deep micropores, fissures, high roughness	High surface area; enhanced adsorption and diffusion
HTC5-25hr (hydrothermal carbonisation)	Spherical aggregates, smoother	Carbon microspheres, shallow pores	Moderate surface area; good functional groups but lower porosity than pyrochar

**Table 3:** Comparison of elemental analysis of the different adsorbent samples.

Adsorbent	C %	H %	N %	Sulphur %	H/C Ratio
PP-Raw	39.77	6.38	2.09	<0.3	1.93
HTC1-2h	54.23	5.27	2.44	-	1.17
HTC1-25h	62.17	5.74	2.85	-	1.11
HTC5-2h	37.09	6.80	1.25	<0.3	2.20
HTC5-25h	53.28	5.24	2.55	<0.3	1.18
PRYR1-1	16.59	1.10	0.32	1.89	0.80
PRYR1-4	13.95	0.66	0.32	2.55	0.57
PRYR2-1	12.36	1.13	<0.3	<0.3	1.10
PRYR2-4	10.02	1.45	<0.3	<0.3	1.74

**Table 4:** Elemental analysis results and BET surface area and pore diameter effects on CBZ adsorption.

Adsorbent	H/C Atomic Ratio	N/C Atomic Ratio	BET Surface Area (m^2^/g)	Pore Diameter (nm)	CBZ % Removal at 1 min
PP-Raw	1.93	0.045	0.12 ± 0.01	5.05	n.d
HTC1-2h	1.17	0.039	0.73 ± 0.03	3.65	74.2
HTC1-25h	1.11	0.039	0.95 ± 0.01	2.60	94.4
HTC5-2h	2.20	0.029	Inconclusive	-	11.9
HTC5-25h	1.18	0.041	1.73 ± 0.04	5.06	97.0
PRYR1-1	0.80	0.017	4.23 ± 0.03	19.77	54.7
PRYR1-4	0.57	0.020	3.77 ± 0.03	14.15	40.7
PRYR2-1	1.10	Nd	0.38 ± 0.01	11.74	23.0
PRYR2-4	1.74	Nd	1.52 ± 0.01	6.25	7.3

**Table 5:** Comparison of CBZ removal capacities of HTC5-25hr and PRYR1-1 with other similar materials from the literature.

Material	Surface Area(m^2^/g)	Q_max_ (mg/g)	Normalized Capacity (mg/m^2^)	Contact Time	Ref.
MTPEE	3.58	0.457	0.13	2.75 h	[63]
HTC5-25h	1.73	47.5	28.03 *	1 min	Current work
PRYR1-1	4.23	27.4	6.32 *	1 min	Current work
MH-HTC-800-1:1	906	190	0.21	ND	[56]
BL700	25.17	0.32	0.0132	168 h	[64]
RH-AC	1408	263	0.187	24 h	[65]
ZCA-CSC	1729	384	0.220	24 h	[65]
BAHC	1265	376	0.297	24 h	[28]

* Uptake based on removal experiments at 50 mg/L and dosage of 1 g/L—not Langmuir capacity.

## Data Availability

Data available upon request.

## Supplementary Materials

The following supporting information can be downloaded at: https://www.mdpi.com/article/10.3390/molecules31132222/s1, Figure S1: BET isotherms for the PRYR samples; Figure S2: BET Isotherms for HTC samples.; Figure S3: Calibration Curve for the determination of CBZ using UV spectrophotometry. UV absorbance all measured at wavelength of nm; Table S1: ANOVA analysis table for the dependence of CBZ removal on the pyrolysis process conditions.; Table S2: ANOVA analysis table for the dependence of CBZ removal on the hydrothermal carbonisation process conditions.

## References

[1] Augustine D., Abdelhaleem A., Ookawara S., Nasr M. (2024). A Novel Adsorption/Co-Digestion/Pyrolysis Scheme for Potato Peel Waste Management to Fulfill the Sustainable Development Goals (SDGs). Waste Biomass Valorizat.

[2] Hidalgo D., Urueña A., Díez D., Martín-Marroquín J.M., Kumar V., Bhat S.A., Verma P., Kumar S. (2024). Hydrothermal Carbonization of Industrial Sludge: Recent Advances, Challenges, and Perspectives. Recent Trends in Management and Utilization of Industrial Sludge.

[3] aus der Beek T., Weber F.A., Bergmann A., Hickmann S., Ebert I., Hein A., Küster A. (2015). Pharmaceuticals in the environment—Global occurrences and perspectives. Environ. Toxicol. Chem..

[4] Biswal B.K., Balasubramanian R. (2022). Adsorptive removal of sulfonamides, tetracyclines and quinolones from wastewater and water using carbon-based materials: Recent developments and future directions. J. Clean. Prod..

[5] Vilakazi S.P., Muchaonyerwa P., Buthelezi-Dube N.N. (2023). Characteristics and liming potential of biochar types from potato waste and pine-bark. PLoS ONE.

[6] Liang S., McDonald A.G. (2014). Chemical and Thermal Characterization of Potato Peel Waste and Its Fermentation Residue as Potential Resources for Biofuel and Bioproducts Production. J. Agric. Food Chem..

[7] Bouchelta C., Medjram M.S., Zoubida M., Chekkat F.A., Ramdane N., Bellat J.-P. (2012). Effects of pyrolysis conditions on the porous structure development of date pits activated carbon. J. Anal. Appl. Pyrolysis.

[8] Osman A.I., Blewitt J., Abu-Dahrieh J.K., Farrell C., Al-Muhtaseb A.a.H., Harrison J., Rooney D.W. (2019). Production and characterisation of activated carbon and carbon nanotubes from potato peel waste and their application in heavy metal removal. Environ. Sci. Pollut. Res..

[9] Si H., Zhao C., Wang B., Liang X., Gao M., Jiang Z., Yu H., Yang Y., Gu Z., Ogino K. (2023). Liquid-solid ratio during hydrothermal carbonization affects hydrochar application potential in soil: Based on characteristics comparison and economic benefit analysis. J. Environ. Manag..

[10] Danso-Boateng E., Ross A.B., Mariner T., Hammerton J., Fitzsimmons M. (2022). Hydrochars produced by hydrothermal carbonisation of seaweed, coconut shell and oak: Effect of processing temperature on physicochemical adsorbent characteristics. SN Appl. Sci..

[11] Taotao S., Mian Muhammad Ahson A., Guangquan C., Yuchen Y., Wentao X., Changsheng P. (2024). Properties of Biochar Prepared by Solar Pyrolysis and Its Adsorption of Cu^2+^ in Water. Earth Sci..

[12] Yao F., Ye G., Peng W., Zhao G., Wang X., Wang Y., Zhu W., Jiao Y., Huang H., Ye D. (2023). Preparation of activated biochar with adjustable pore structure by hydrothermal carbonization for efficient adsorption of VOCs and its practical application prospects. J. Environ. Chem. Eng..

[13] Goyi A.A., Sher Mohammad N.M., Omer K.M. (2024). Preparation and characterization of potato peel derived hydrochar and its application for removal of Congo red: A comparative study with potato peel powder. Int. J. Environ. Sci. Technol..

[14] Köchermann J., Görsch K., Wirth B., Mühlenberg J., Klemm M. (2018). Hydrothermal carbonization: Temperature influence on hydrochar and aqueous phase composition during process water recirculation. J. Environ. Chem. Eng..

[15] Román S., Nabais J.M.V., Laginhas C., Ledesma B., González J.F. (2012). Hydrothermal carbonization as an effective way of densifying the energy content of biomass. Fuel Process. Technol..

[16] Guo Q., Qiao S., Zhang D., Zhang Z., Yu F., Ma Z., Hu Y. (2024). A comparison of hydrothermal carbonization versus pyrolysis-activation for sludge-derived carbon materials on physiochemical properties and electrochemical performance. Biomass Bioenergy.

[17] Patel M., Kumar R., Kishor K., Mlsna T., Pittman C.U., Mohan D. (2019). Pharmaceuticals of Emerging Concern in Aquatic Systems: Chemistry, Occurrence, Effects, and Removal Methods. Chem. Rev..

[18] Fent K., Weston A.A., Caminada D. (2006). Ecotoxicology of human pharmaceuticals. Aquat. Toxicol..

[19] Décima M.A., Marzeddu S., Barchiesi M., Di Marcantonio C., Chiavola A., Boni M.R. (2021). A Review on the Removal of Carbamazepine from Aqueous Solution by Using Activated Carbon and Biochar. Sustainability.

[20] Liu Y., Li Q., Pan H., Liang L., Zhao L., Shi Q., Zhao C., Liu X. (2025). Adsorption of carbamazepine on self-endowed magnetic biochar produced from iron-rich sludge. RSC Adv..

[21] Agilandeswari P., Venkateshbabu S., Sarojini G., Rajasimman M. (2023). Sustainable development and analysis of a novel bio-derived (biochar) nanocomposite for the remediation of carbamazepine from aqueous solution. Chemosphere.

[22] Saqib N.U., Naqvi M., Li B., Munir M.T., Zhong H., Wang Z., Zhu Z. (2024). Utilizing hydrothermally carbonized food waste-derived activated hydrochar for the elimination of carbamazepine and naproxen. Res. Sq..

[23] Stoykova M., Koumanova B., Mörl L. (2013). Adsorptive removal of carbamazepine from wastewaters by activated charcoals. J. Chem. Technol. Metall..

[24] Campbell R., Xiao B., Mangwandi C. (2024). Production of activated carbon from spent coffee grounds (SCG) for removal of hexavalent chromium from synthetic wastewater solutions. J. Environ. Manag..

[25] Harimisa G.E., Jusoh N.W.C., Tan L.S., Shameli K., Ghafar N.A., Masudi A. (2022). Synthesis of potassium hydroxide-treated activated carbon via one-step activation method. J. Phys. Conf. Ser..

[26] Thunshirn P., Wenzel W.W., Pfeifer C. (2022). Pore characteristics of hydrochars and their role as a vector for soil bacteria: A critical review of engineering options. Crit. Rev. Environ. Sci. Technol..

[27] Yukhymchuk A., Zhukova D., Prybora N., Stolyarchuk N., Bondarchuk O., Bodnár Yankovych H., Melnyk I.V. (2024). Waste-to-Wealth: Unlocking the Potential of Pine Sawdust Biochar for Adsorption of Cobalt(II) and Nickel(II) Ions and Sustainable Elimination of Carbamazepine from Aqueous Solutions. ACS EST Water.

[28] Zhong H., Zhu G., Wang Z., Liu X., Zhang H., Qiu Y., Yin D., Zhu Z. (2025). Efficient adsorption removal of carbamazepine from water by dual-activator modified hydrochar. Sep. Purif. Technol..

[29] Zhang W., Sheng X., Yan J., Wang J., Sun J., Zuo Q., Zhu X., Wang M., Gong L. (2023). The crucial role of different NaOH activation pathways on the algae-derived biochar toward carbamazepine adsorption. Results Eng..

[30] Kyotani M., Matsushita S., Kimura S.-i., Akagi K. (2012). Efficient preparation of carbon papers by pyrolysis of iodine-treated Japanese paper. J. Anal. Appl. Pyrolysis.

[31] Castillo K.J.T., Detras M.C.M., Alfafara C.G., Migo V.P. (2024). Optimization study for chemical activation of biochar derived from sugarcane bagasse for maximum phytohormone adsorption from waste coconut water. IOP Conf. Ser. Mater. Sci. Eng..

[32] Huang Y., Guan Y., Dong W., Wang Y., Wang P., Wu M., Yi P., Chen Q., Pan B. (2025). Understanding the pore sorption of antibiotics by carbon-based materials: Integrating experimental and computational approach. Chem. Eng. Sci..

[33] Khosravi A., Zheng H., Liu Q., Hashemi M., Tang Y., Xing B. (2022). Production and characterization of hydrochars and their application in soil improvement and environmental remediation. Chem. Eng. J..

[34] Kozyatnyk I., Benavente V., Weidemann E., Gentili F.G., Jansson S. (2023). Influence of hydrothermal carbonization conditions on the porosity, functionality, and sorption properties of microalgae hydrochars. Sci. Rep..

[35] Wortmann M., Keil W., Diestelhorst E., Westphal M., Haverkamp R., Brockhagen B., Biedinger J., Bondzio L., Weinberger C., Baier D. (2023). Hard carbon microspheres with bimodal size distribution and hierarchical porosity via hydrothermal carbonization of trehalose. RSC Adv..

[36] Kozyatnyk I., Yakupova I. (2025). Impact of Chemical and Physical Treatments on the Structural and Surface Properties of Activated Carbon and Hydrochar. ACS Sustain. Chem. Eng..

[37] Mehrabinia P., Ghanbari-Adivi E., Fattahi R., Samimi H.A., Kermanezhad J. (2022). Nitrate removal from agricultural effluent using sugarcane bagasse active nanosorbent. J. Appl. Water Eng. Res..

[38] Sufian J., Babaakbari Sari M., Marchelli F., Fiori L., Avanes A., Moradi S. (2023). Algal Biomass, Biochar and Hydrochar from Chlorella Vulgaris for Cadmium Removal from Aqueous Streams. Res. Sq..

[39] Kumar Mishra R., Singh B., Acharya B. (2024). A comprehensive review on activated carbon from pyrolysis of lignocellulosic biomass: An application for energy and the environment. Carbon Resour. Convers..

[40] Malini K., Selvakumar D., Kumar N.S. (2023). Activated carbon from biomass: Preparation, factors improving basicity and surface properties for enhanced CO2 capture capacity—A review. J. CO2 Util..

[41] Putra A.E.E., Amaliyah N., Syam M., Rahim I. (2019). Effect of Residence Time and Chemical Activation on Pyrolysis Product from Tires Waste. J. Jpn. Inst. Energy.

[42] Yang X., Kwon E.E., Dou X., Zhang M., Kim K.-H., Tsang D.C.W., Ok Y.S. (2018). Fabrication of spherical biochar by a two-step thermal process from waste potato peel. Sci. Total Environ..

[43] Smith A.M., Ross A.B. (2019). The Influence of Residence Time during Hydrothermal Carbonisation of Miscanthus on Bio-Coal Combustion Chemistry. Energies.

[44] Wang G., Liu J., Liang W., Dan J., Ning X., Wang C. (2023). Hydrothermal carbonization mechanism of agricultural waste under different conditions: An experimental and ReaxFF molecular dynamics study. J. Energy Inst..

[45] Aragón-Briceño C.I., Pozarlik A.K., Bramer E.A., Niedzwiecki L., Pawlak-Kruczek H., Brem G. (2021). Hydrothermal carbonization of wet biomass from nitrogen and phosphorus approach: A review. Renew. Energy.

[46] Su X., He J., Khan M.A., Chang K., Liu Y., Guo G., Li X., Jin F., Kuang M., Gouda S. (2023). Potential Application Performance of Hydrochar from Kitchen Waste: Effects of Salt, Oil, Moisture, and pH. Toxics.

[47] Ganesapillai M., Mehta R., Tiwari A., Sinha A., Bakshi H.S., Chellappa V., Drewnowski J. (2023). Waste to energy: A review of biochar production with emphasis on mathematical modelling and its applications. Heliyon.

[48] El-Azazy M., El-Shafie A.S., Issa A.A., Al-Sulaiti M., Al-Yafie J., Shomar B., Al-Saad K. (2019). Potato Peels as an Adsorbent for Heavy Metals from Aqueous Solutions: Eco-Structuring of a Green Adsorbent Operating Plackett–Burman Design. J. Chem..

[49] Farooq U., Khan M.A., Athar M., Kozinski J.A. (2011). Effect of modification of environmentally friendly biosorbent wheat (Triticum aestivum) on the biosorptive removal of cadmium(II) ions from aqueous solution. Chem. Eng. J..

[50] Bodîrlău R., Teacă C.A., Spiridon I. (2009). Preparation and characterization of composites comprising modified hardwood and wood polymers/poly (vinyl chloride). BioResources.

[51] Johari K., Saman N., Song S.T., Mat H. (2015). Adsorption equilibrium and kinetics of elemental mercury onto coconut pith. J. Environ. Sci. Technol..

[52] Van Soest J.J., Tournois H., de Wit D., Vliegenthart J.F. (1995). Short-range structure in (partially) crystalline potato starch determined with attenuated total reflectance Fourier-transform IR spectroscopy. Carbohydr. Res..

[53] Kyzas G.Z., Deliyanni E.A. (2015). Modified activated carbons from potato peels as green environmental-friendly adsorbents for the treatment of pharmaceutical effluents. Chem. Eng. Res. Des..

[54] Arampatzidou A.C., Deliyanni E.A. (2016). Comparison of activation media and pyrolysis temperature for activated carbons development by pyrolysis of potato peels for effective adsorption of endocrine disruptor bisphenol-A. J. Colloid Interface Sci..

[55] Tran N.H., Reinhard M., Gin K.Y. (2018). Occurrence and fate of emerging contaminants in municipal wastewater treatment plants from different geographical regions—A review. Water Res..

[56] Apinyakul N., Chanpee S., Kaewtrakulchai N., Khemasiri N., Eiad-ua A., Assawasaengrat P. (2024). Synthesis of nanoporous carbon from brewer waste by hydrothermal carbonization assisted chemical activation for carbamazepine adsorption. Case Stud. Chem. Environ. Eng..

[57] Chen D., Xie S., Chen C., Quan H., Hua L., Luo X., Guo L. (2017). Activated biochar derived from pomelo peel as a high-capacity sorbent for removal of carbamazepine from aqueous solution. RSC Adv..

[58] Wang B., Jiang Y.-S., Li F.-Y., Yang D.-Y. (2017). Preparation of biochar by simultaneous carbonization, magnetization and activation for norfloxacin removal in water. Bioresour. Technol..

[59] Mäkelä M., Benavente V., Fullana A. (2015). Hydrothermal carbonization of lignocellulosic biomass: Effect of process conditions on hydrochar properties. Appl. Energy.

[60] Ahmed M.B., Zhou J.L., Ngo H.H., Guo W., Chen M. (2016). Progress in the preparation and application of modified biochar for improved contaminant removal from water and wastewater. Bioresour. Technol..

[61] Li J., Li Y., Xiong Z., Yao G., Lai B. (2019). The electrochemical advanced oxidation processes coupling of oxidants for organic pollutants degradation: A mini-review. Chin. Chem. Lett..

[62] Pezoti O., Cazetta A.L., Souza I.P.A.F., Bedin K.C., Martins A.C., Silva T.L., Almeida V.C. (2014). Adsorption studies of methylene blue onto ZnCl2-activated carbon produced from buriti shells (*Mauritia flexuosa* L.). J. Ind. Eng. Chem..

[63] Mahdavi P., Siol A., Thöming J. (2026). Removal of carbamazepine and diclofenac at trace levels from water using a regenerable polymeric adsorbent and a desorption-based analytical method. J. Water Process Eng..

[64] Chu G., Zhao J., Liu Y., Lang D., Wu M., Pan B., Steinberg C.E.W. (2019). The relative importance of different carbon structures in biochars to carbamazepine and bisphenol A sorption. J. Hazard. Mater..

[65] Lilli B., Wassersleben S., Schulze T., Otto A., Enke D. (2024). Additive effects of rice husk-based carbon-silica composites on adsorption of diclofenac sodium and carbamazepine from aqueous solutions. Sci. Total Environ..

[66] Blankenship L.S., Jagiello J., Mokaya R. (2022). Confirmation of pore formation mechanisms in biochars and activated carbons by dual isotherm analysis. Mater. Adv..

[67] Chen B., Guan H., Zhang Y., Liu S., Zhao B., Zhong C., Zhang H., Ding W., Song A., Zhu D. (2023). Performance and mechanism of Pb^2+^ and Cd^2+^ ions’ adsorption via modified antibiotic residue-based hydrochar. Heliyon.

[68] Dieguez-Alonso A., Funke A., Anca-Couce A., Rombolà A.G., Ojeda G., Bachmann J., Behrendt F. (2018). Towards Biochar and Hydrochar Engineering—Influence of Process Conditions on Surface Physical and Chemical Properties, Thermal Stability, Nutrient Availability, Toxicity and Wettability. Energies.

[69] Vuppaladadiyam A.K., Jena M.K., Hakeem I.G., Patel S., Veluswamy G., Thulasiraman A.V., Surapaneni A., Shah K. (2024). A critical review of biochar versus hydrochar and their application for H2S removal from biogas. Rev. Environ. Sci. Bio/Technol..

[70] Huang Z., Campbell R., Mangwandi C. (2024). Kinetics and Thermodynamics Study on Removal of Cr(VI) from Aqueous Solutions Using Acid-Modified Banana Peel (ABP) Adsorbents. Molecules.

[71] Han J., Albadarin A.B., Guo C., Annath H., Mangwandi C. (2026). Solubility enhancement of BCS class II drugs via in-situ loading onto MIL-101(Cr) in a green solvent system. Sep. Purif. Technol..

[72] Jaiyeola O.O., Annath H., Mangwandi C. (2023). Synthesis and evaluation of a new CeO_2_@starch nanocomposite particles for efficient removal of toxic Cr(VI) ions. Energy Nexus.

