# Supplementary material for: Efficient Removal of Carbamazepine from Synthetic Wastewater Using Potato Peel-Derived Hydrochars: A Comparative Study of Hydrothermal and Pyrolytic Conversion
Source: Molecules. 2026 Jun 24;31(13):2222. doi: 10.3390/molecules31132222 (PMC13363086; doi:10.3390/molecules31132222)
Supplement: Supplementary file 1 [file molecules-31-02222-s001.zip › molecules-4303430-supplementary.pdf]

Efficient Removal of Carbamazepine from Synthetic Wastewater Using Potato Peel-Derived Hydro-chars: A Comparative Study of Hydrothermal and Pyrolytic Conversion

Justin Khong, Bo Xiao and Chirangano Mangwandi*

School of Chemistry & Chemical Engineering, Queen's University Belfast, David Kier Building, Stranmillis Road, Belfast, BT95AG, United Kingdom

Corresponding author email: mailto:c.mangwandi@qub.ac.uk c.mangwandi@qub.ac.uk

Supplementary Information

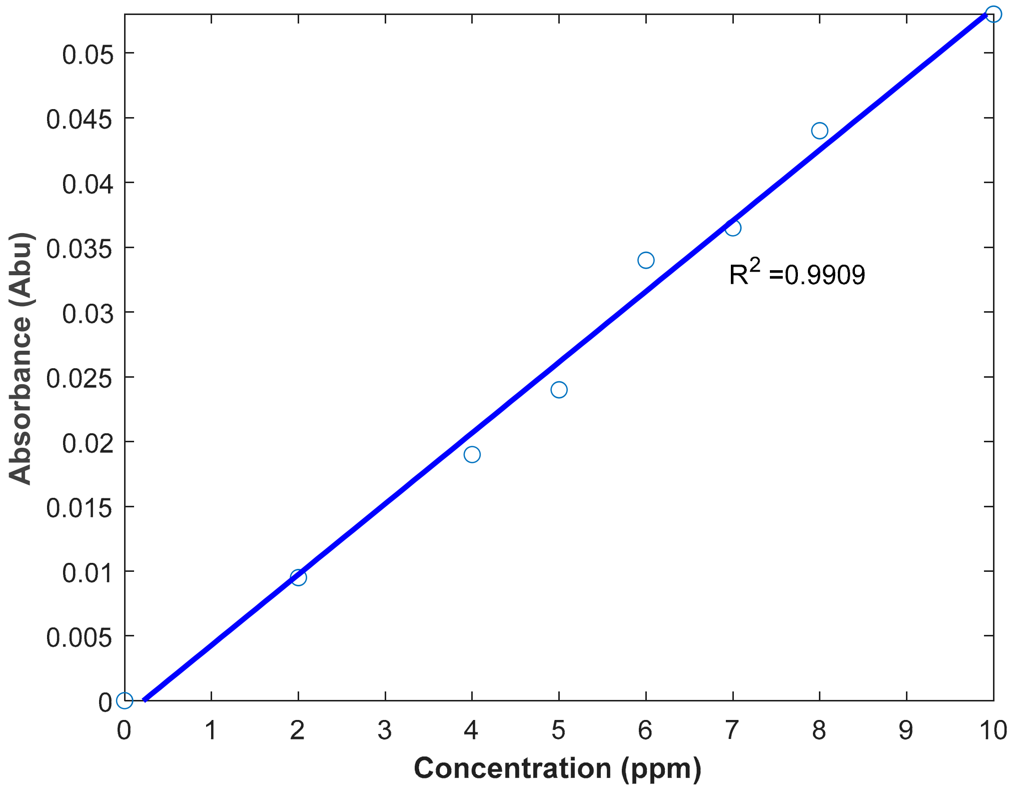

Figure S. 1 . Calibration Curve for the determination of CBZ using UV spectrophotometry. UV absorbance all measured at wavelength of nm.

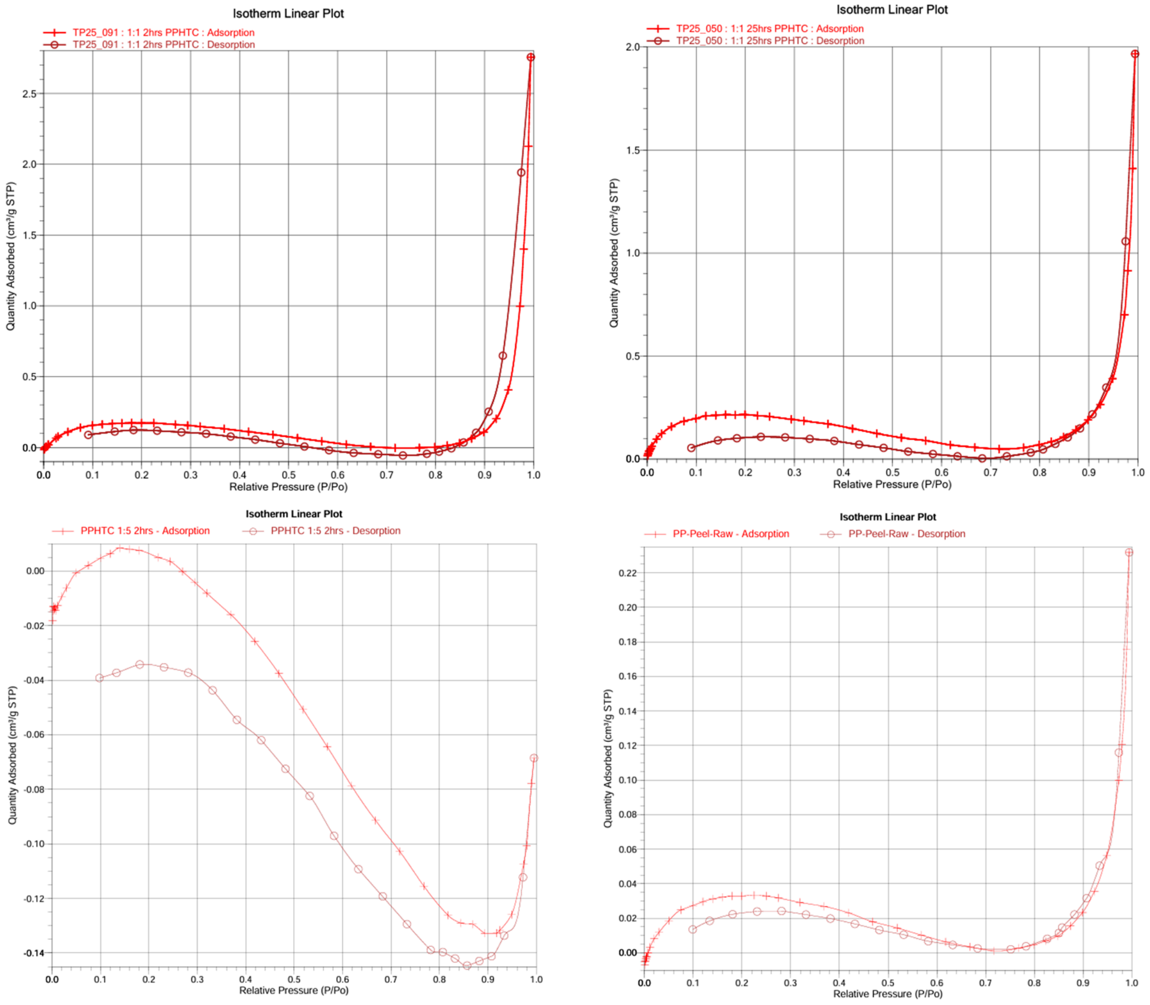

Figure S. 2: BET Isotherms for HTC samples.

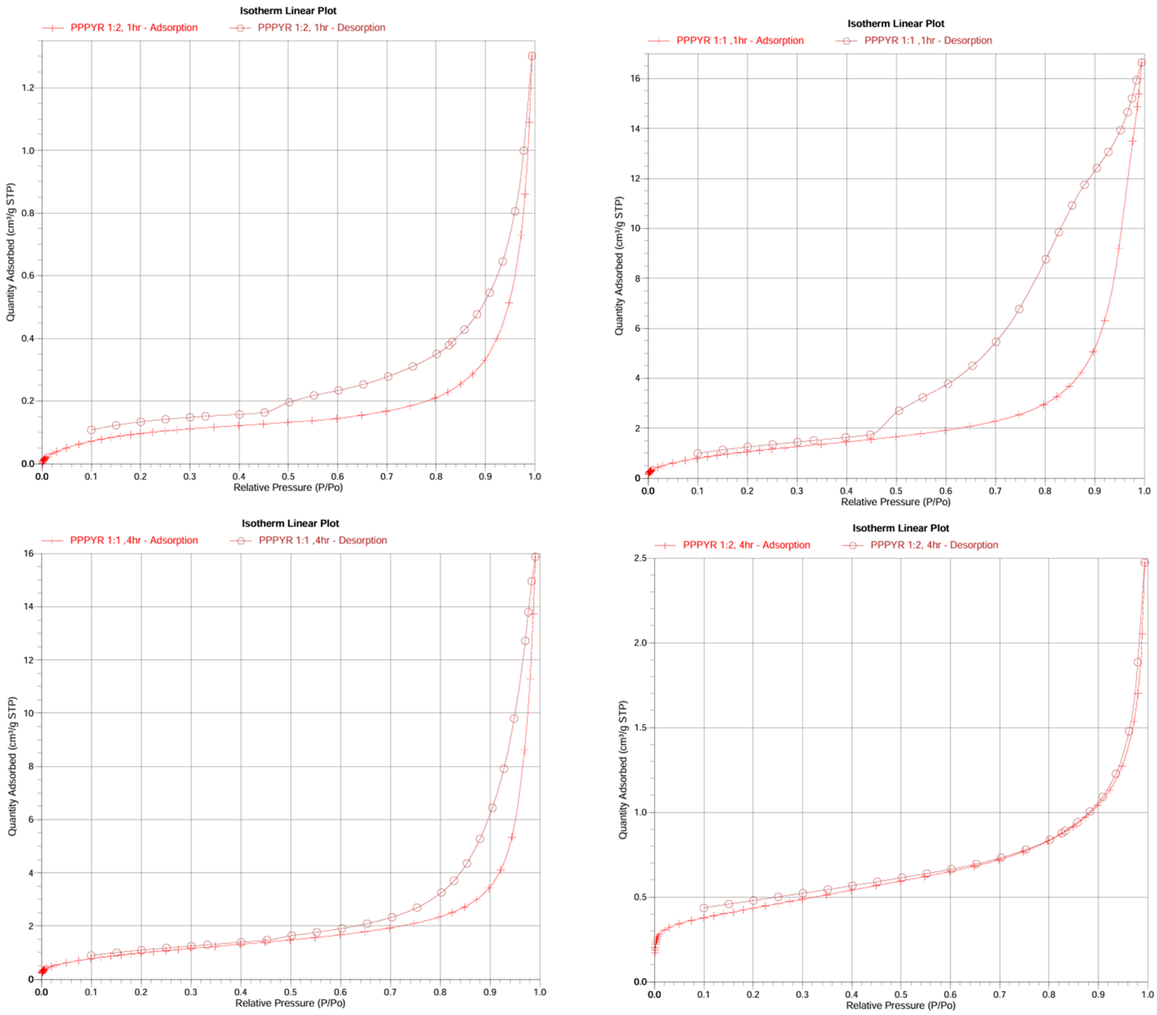

Figure S. 3. BET isotherms for the PRYR samples.

Table S. 1. ANOVA analysis table for the dependence of CBZ removal on the pyrolysis process conditions.

Source	Sum Sq.	d.f.	Mean Sq.	F	Prob>F
x_1	1059.5	1	1059.5	1466.44	0.0166
x_2	220.52	1	220.52	350.22	0.0064
Error	0.72	1	0.72		
Total	1280.75	3			

NB. X1 is KOH: Biomass mass ratio; x2 is pyrolysis time

Table S. 2.ANOVA analysis table for the dependence of CBZ removal on the hydrothermal carbonisation process conditions

Source	Sum Sq.	d.f.	Mean Sq.	F	Prob>F
x_1	832.32	1	832.32	0.84	0.5274
x_2	2878.32	1	2878.32	2.91	0.3375
Error	989.1	1	989.1		
Total	4699.75	3			

NB. X1 is Water: Biomass mass ratio; x2 is reaction time
